# The Potential Role of the Posterior Elements in Lumbar Spine Laminoplasty to Protect the Intervertebral Disc and Improve Walking Ability—Retrospective Comparative Study

**DOI:** 10.3390/jcm14124014

**Published:** 2025-06-06

**Authors:** Namito Nakashita, Takashi Ohnishi, Tomomichi Kajino, Yuichiro Hisada, Hideki Sudo, Katsuhisa Yamada, Tsutomu Endo, Daisuke Ukeba, Yuichi Hasegawa, Toshiya Chubachi, Norimasa Iwasaki

**Affiliations:** 1 Department of Orthopaedic Surgery, Hokkaido University Hospital, N15W7, Sapporo 060-8638, Hokkaido, Japan; 2 Department of Orthopaedic Surgery, Tonan Hospital, N4W7, Sapporo 060-0004, Hokkaido, Japan; 3 Department of Orthopaedic Surgery, Faculty of Medicine and Graduate School of Medicine, Hokkaido University, N15W7, Sapporo 060-8638, Hokkaido, Japan

**Keywords:** lumbar spine, laminoplasty, posterior elements, intervertebral disc degeneration, walking ability

## Abstract

**Objectives**: To investigate whether preservation of the posterior elements protects the spine from degeneration and improves postoperative symptoms in lumbar spine laminoplasty. **Methods**: Eighty-five consecutive patients who underwent lumbar spine laminoplasty were retrospectively reviewed. They were non-randomly stratified into two groups, the posterior elements resection (R) group and the preservation (P) group, and they were followed for two years after surgery. We radiographically analyzed the conditions of the spine and intervertebral disc (IVD) two years after surgery. The Japanese Orthopaedic Association Back Pain Evaluation Questionnaire (JOABPEQ) was used for symptom assessments. Logistic regression analysis was performed to determine whether the kissing spine was a significant factor for the outcomes in group R. **Results**: The 2-year D score increment and 2-year IVD height decrement was lower in group P. No difference was found in the flexion–extension angles or incidence of instability between groups. The JOABPEQ revealed higher scores in walking ability, social life function, and mental health in group P one year after surgery. Walking ability was the only score that remained higher two years after surgery. The visual analog scale of pain in the buttocks and lower limbs was lower in group P only one year after surgery. Finally, the kissing spine was not a significant factor in any outcome. **Conclusions**: The preserved posterior elements were considered to protect the IVD in lumbar spine laminoplasty. In addition, they positively affected postoperative health status from multiple aspects.

## 1. Introduction

Lumbar canal stenosis (LCS) is caused by narrowing of the vertebral canal involving sciatica and/or peripheral sensational disorders, intermittent claudication, and bladder bowel disorder in the severest condition [1]. LCS occurs after the 40th to 50th year of life and is the primary cause of lumbar spine diseases that require operative treatment after 65 years [1]. The standard surgical technique to treat LCS is laminoplasty [2]. The posterior approach involved in this technique is safer than approaches from other directions, namely, a lack of critical vasculatures, organs, and nervous plexus. However, it often requires the detachment of the paravertebral muscles from the spinous processes and the laminas and resection of the construct of supra- and interspinous ligaments and spinous processes, i.e., “the posterior elements [2,3]”. Preservation of the paravertebral muscle has been highlighted for a decade to mitigate postoperative degeneration of the muscle and lower back pain [2,4]. However, the posterior elements have not been explored clinically. Currently, the available evidence is based on a cadaveric study and an in silico study, which have elucidated the disadvantages of sacrificing the posterior elements [3,5]. Abumi et al. reported that only the flexion range of motion increased after resecting the posterior elements in the lumbar spine of cadavers [3]. An in silico study using finite element analysis yielded similar results, showing the largest increase in the flexion range of motion in the L4/5 level followed by the L3/4 level [5]. However, a clinical study to demonstrate the role of the posterior elements in regulating the physiological flexion range of motion at the surgical site is absent. Moreover, whether preservation of the posterior elements protects the intervertebral disc (IVD) at the surgical level or improves postoperative symptoms is unknown.

The objectives of the present study are to investigate whether preserving the posterior elements protects the IVD from degeneration and improves postoperative symptoms in lumbar spine laminoplasty. Herein, we unravel the role of the posterior elements in this wide-spread surgical technique from a “bench to bedside” translational point of view, casting an important lesson on the evidence of spine surgery.

## 2. Materials and Methods

We obtained ethical approval from the Research Ethics Board of the author’s affiliated institution (# 022-0219). The present study is registered to the University hospital Medical Information Network (UMIN). The inclusion criteria were cases which underwent laminoplasty for lumbar spinal canal stenosis without segmental instability, which is defined by 3 mm > of translation in flexion–extension radiographs. The exclusion criteria were cases which underwent laminoplasty for degenerative scoliosis with a Cobb angle > 20 degrees, osteolytic spinal diseases, and adjacent segmental disease secondary to spinal fusion surgeries. Eighty-five consecutive patients who underwent single- or multiple-level lumbar spine laminoplasty were reviewed retrospectively. They were non-randomly stratified into two groups, the posterior elements resection (R) group and the preservation (P) group, in which the posterior elements indicate the construct of supra- and interspinous ligaments and spinous processes.

**Figure 1:**
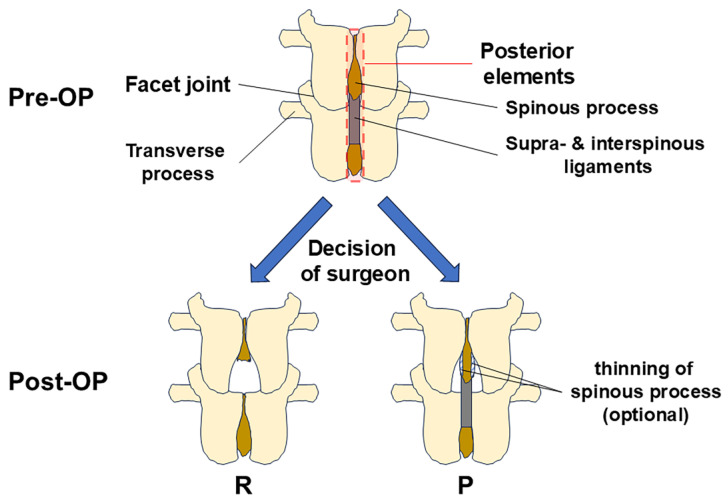
Flow of decisions and illustration of the surgical techniques of the posterior elements resection (R) group and the preservation (P) group. OP, operation.

We will explain the surgical techniques step-by-by (Figure 1). After detaching paravertebral muscles and exposing the spine, laminoplasty was performed with or without resecting the posterior elements (Figure 1). Adequate decompression can be achieved by medial facetectomy and flavectomy from both sides, preserving the posterior elements (Figure 1). The interspinous ligament should be preserved by identifying its borderline between the ligamentum flavum. We defined that thinning of the spinous processes to improve surgical visibility did not breach the functional integrity of the posterior elements, since the longitudinal continuity of the tissue was intact (Figure 1).

Selection of whether or not to preserve the posterior elements was based on the decision of surgeons. Three of the eleven surgeons involved had a hypothetic principle that preserving the posterior elements during laminoplasty might protect the postoperative stability of the operated spinal segment. Therefore, those three surgeons preserved the elements unless it was technically demanding, depending on the cases.

The patients were followed for two years after surgery. Radiographic analyses included a postoperative 2-year Kelgren and Lawrence (KL) score [6,7] increment, 2-year D score [8] increment, 2-year IVD height (DH) decrement, and an increase in active flexion–extension angles at the surgical levels. Briefly, KL scores consist of grade 0, indicating absence of degeneration, to grade 4, indicating severe degeneration in the spine [6,7]. The D score is a grading system of the IVD part of the KL score with modifications; it ranges from a score of 0, indicating intact, to a score of 3, indicating collapse and/or sclerosis of the vertebral endplates [8]. In the present study, a single established spine surgeon rated all the radiographic measures; therefore, evaluation of inter-observer reliability does not apply. Logistic regression analyses or multiple regression analysis were performed to assess whether surgical group was significant for the 2-year KL score increment, D score increment, and DH decrement adjusting for surgical level, age, sex, each comorbidity, and smoking history. We determined the occurrence or deterioration (incidence) of instability at the surgical level two years after surgery. The Japanese Orthopaedic Association Back Pain Evaluation Questionnaire (JOABPEQ) [9] was used for symptom-based disability assessments before surgery and one and two years after surgery in single-level laminoplasty. Logistic regression analysis was performed to determine whether the kissing spine was a significant factor for the radiographic and symptom-based disability outcomes. Surgical time, intraoperative hemorrhage, and levels of change in laboratory data within the postoperative seven days were compared between groups in single-level laminoplasty. Student’s t-test, Wilcoxon’s test, or Fisher’s exact test were used to compare the data between groups depending on the results of the Shapiro–Wilk test when it was applicable. The results of the Shapiro–Wilk test are reported in Appendix A. Due to the exploratory nature of the present study, we did not perform *p*-value adjustment for multiple comparisons. Due to missing data depending on the analyses, we indicated the numbers of data in each subsection of the Results section.

## 3. Results

### 3.1. Demographic Background

The ages (*p* = 0.31), gender ratios (*p* = 1.00), prevalence of major comorbidities (*p* = 0.45–0.66), and ratios of smoking history (*p* = 0.82) were similar between group R (n = 38) and group P (n = 47) (Table 1). The investigated comorbidities were diabetes mellitus (*p* = 0.47), hypertension (*p* = 0.66), chronic kidney disease (*p* = 0.50), and rheumatoid arthritis (*p* = 0.45) (Table 1). Operated spinal levels were comparable between groups (*p* = 0.06) (Table 1). The L4/5 level was more operated on and the L2/3 level was less operated on in group P compared to group R, but there was no statistical difference.

### 3.2. Surgical Performance and Perioperative Laboratory Data in Single-Level Laminoplasty

To analyze the surgical performance and perioperative laboratory data relevant to surgical invasiveness depending on the surgical procedures, we compared those of single-level laminoplasty between groups.

**Figure 2:**
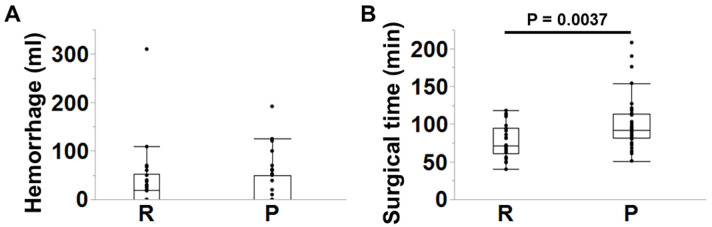
Comparisons of intraoperative (**A**) hemorrhage and (**B**) surgical time between the posterior elements resection (R) group and the preservation (P) group. Hemorrhage: group R:P, n = 26:37; *p* = 0.34; surgical time: group R:P, n = 26:37; *p* = 0.0037. Error bars indicate standard deviation. Wilcoxon’s tests were performed due to nonparametric data distribution.

**Figure 3:**
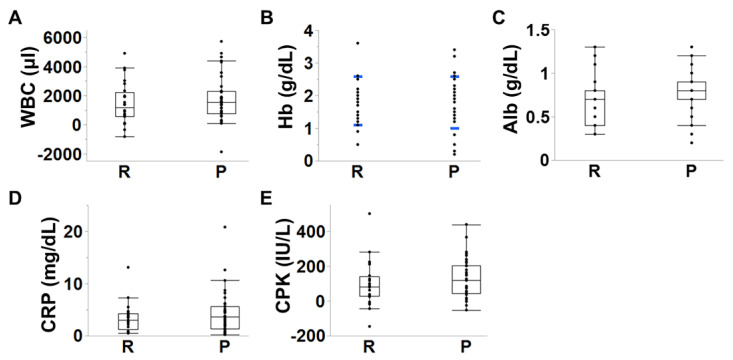
Comparisons of change in laboratory data relevant to surgical invasiveness between the posterior elements resection (R) group and the preservation (P) group. (**A**) WBC, white blood cell counts (*p* = 0.41); (**B**) Hb, hemoglobin (*p* = 0.80); (**C**) Alb, albumin (*p* = 0.12); (**D**) CRP, C-reactive protein (*p* = 0.48); (**E**) CPK, creatine phosphokinase (*p* = 0.24) within the postoperative seven days (Group R:P, n = 23–25:32–37). Student’s *t*-tests were performed only for the Hb. Error bars indicate standard deviation. The others were tested by the Wilcoxon tests.

The estimated intraoperative blood loss (hemorrhage) was equivalent between groups (group R:P, n = 26:37; *p* = 0.34) (Figure 2A). However, surgical time was significantly longer in group P (group R:P, n = 26:37; *p* = 0.004) (Figure 2B). There was no difference in the changes in laboratory data relevant to surgical invasiveness, such as white blood cell counts (*p* = 0.41), hemoglobin (*p* = 0.80), albumin (*p* = 0.12), C-reactive protein (*p* = 0.48), and creatine phosphokinase (*p* = 0.24) within the postoperative seven days (group R:P, n = 23–25:32–37) (Figure 3A–E).

The complications and revisions were as follows: there was an inferior articular process fracture in one case, a hematoma requiring surgery in one case, herniation of the IVD in the operated level in one case, instability in the operated level three years after surgery and fusion surgery in one case, surgical site infection requiring debridement and irrigation in one case, and a dura mater tear in one case in group R; there was a relapse of stenosis requiring re-laminoplasty in one case, dehiscence of the wound requiring suturing in one case, hematoma requiring surgery in one case and not requiring surgery in one case, an inferior articular process fracture in one case, herniation of the IVD treated conservatively in two cases, and a dura mater tear in two cases in group P.

### 3.3. Radiographic Outcomes

**Figure 4:**
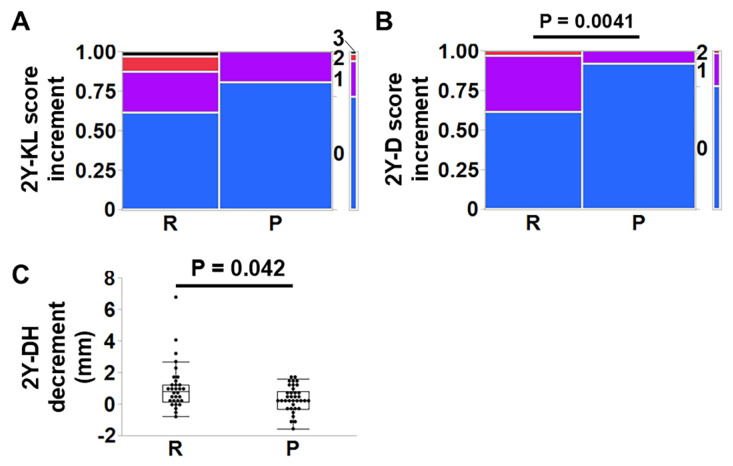
Comparisons of radiographic outcome between the posterior elements resection (R) group and the preservation (P) group. (**A**) The 2-year (Y) Kelgren and Lawrence (KL) score increment (*p* = 0.083), (**B**) 2-year (Y) D score increment (*p* = 0.0041), and (**C**) 2-year (Y) intervertebral disc height (DH) decrement were evaluated (*p* = 0.042) (group R:P, n = 31:36 for the KL score, D score, and DH). Fisher’s exact tests were used for comparisons of KL scores and D scores. The Wilcoxon tests were performed for comparisons of DHs.

**Figure 5:**
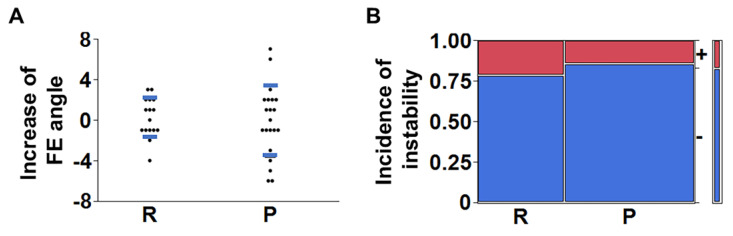
Comparisons of radiographic outcome between the posterior elements resection (R) group and the preservation (P) group. (**A**) The increase in flexion–extension (FE) angles (group R:P, n = 16:21; *p* = 0.64) and (**B**) the incidence of instability (group R:P, n = 14:21; *p* = 0.66) at the surgical levels between groups two years after surgery were investigated. Student’s *t*-tests for the FE angle and Fisher’s exact tests for the incidence of instability were used. Error bars indicate standard deviation in (**A**).

The 2-year KL score increment of group P was slightly lower (*p* = 0.083) (Figure 4A). The 2-year D score increment and 2-year DH decrement were lower in group P (*p* = 0.0041 and 0.042, respectively) (Figure 4B,C) (group R:P, n = 31:36 for the KL score, D score, and DH). In addition, logistic regression analyses or multiple regression analysis were performed to assess whether surgical group was significant for the 2-year KL score increment, D score increment, and DH decrement adjusting for surgical level, age, sex, each comorbidity, and smoking history. As a result, surgical group was a relevant parameter for the 2-year KL score increment (*p* = 0.0073), D score increment (*p* = 0.0017), and DH decrement (*p* = 0.0443). No difference was found in the increase in flexion–extension angles at the surgical levels two years after surgery (group R:P, n = 16:21; *p* = 0.64) (Figure 5A). Similarly, there was no difference in the incidence of instability at the surgical levels between groups two years after surgery (group R:P, n = 14:21; *p* = 0.66) (Figure 5B).

### 3.4. Symptom-Based Assessments

Similar to the analyses of the surgical performance and perioperative laboratory data, we compared the JOABPEQ scores and pain VAS values of single-level laminoplasty cases.

**Figure 6:**
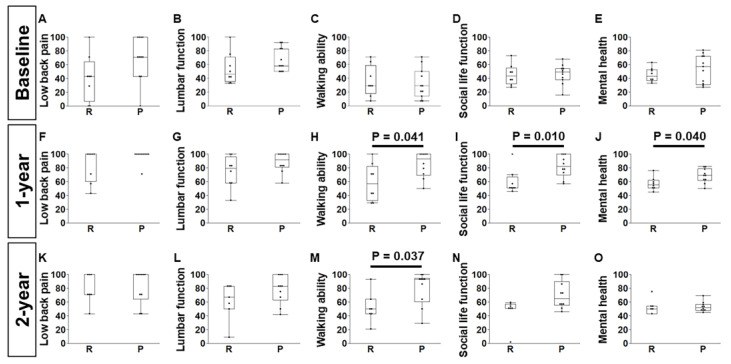
Comparisons of JOABPEQ scores between the posterior elements resection (R) group and the preservation (P) group of single-level laminoplasty cases. Baseline: (**A**) lower back pain (*p* = 0.07), (**B**) lumbar function (*p* = 0.10), (**C**) walking ability (*p* = 0.71), (**D**) social life function (*p* = 0.65), and (**E**) mental health (*p* = 0.48) (group R:P, n = 8:11). One year after surgery: (**F**) lower back pain (*p* = 0.16), (**G**) lumbar function (*p* = 0.16), (**H**) walking ability (*p* = 0.041), (**I**) social life function (*p* = 0.010), and (**J**) mental health (*p* = 0.040) (group R:P, n = 8:10). Two years after surgery: (**K**) lower back pain (*p* = 0.71), (**L**) lumbar function (*p* = 0.23), (**M**) walking ability (*p* = 0.037), (**N**) social life function (*p* = 0.054), and (**O**) mental health (*p* = 0.81) (group R:P, n = 7:10). Wilcoxon’s tests were used.

All the baseline JOABPEQ scores were similar, i.e., lower back pain (*p* = 0.07), lumbar function (*p* = 0.10), walking ability (*p* = 0.71), social life function (*p* = 0.65), and mental health (*p* = 0.48) (group R:P, n = 8:11) (Figure 6A–E). The JOABPEQ scores revealed higher walking ability, social life function, and mental health in group P one year after surgery (group R:P, n = 8:10; *p* = 0.041, 0.010, and 0.040, respectively) (Figure 6H–J), while walking ability was the only one that remained higher at the two-year time point (group R:P, n = 7:10; *p* = 0.037) (Figure 6M). No significant difference was observed in lower back pain or lumbar function one year after surgery (group R:P, n = 8:10; *p* = 0.16 and *p* = 0.16, respectively) (Figure 6F,G). Lower back pain (*p* = 0.71), lumbar function (*p* = 0.23), social life function (*p* = 0.054), and mental health (*p* = 0.81) scores were similar between groups two years after surgery (group R:P, n = 7:10) (Figure 6K,L,N,O).

**Figure 7:**
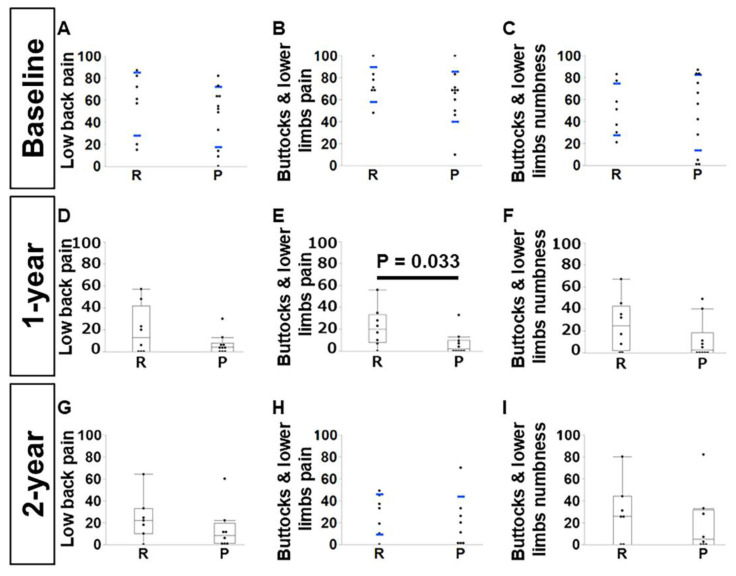
Comparisons of pain visual analog scale between the posterior elements resection (R) group and the preservation (P) group of single-level laminoplasty cases. Baseline: (**A**) lower back pain (*p* = 0.41), (**B**) pain in the buttocks and lower limbs (*p* = 0.28), and (**C**) numbness in the buttocks and lower limbs (*p* = 0.84) (group R:P, n = 7:11); one year after surgery: (**D**) lower back pain (*p* = 0.39), (**E**) pain in the buttocks and lower limbs (*p* = 0.033), and (**F**) numbness in the buttocks and lower limbs (*p* = 0.20) (group R:P, n = 8:10); two years after surgery: (**G**) lower back pain (*p* = 0.22), (**H**) pain in the buttocks and lower limbs (*p* = 0.53), and (**I**) numbness in the buttocks and lower limbs (*p* = 0.60) (group R:P, n = 7:8). (**A**–**C**,**H**) were tested by Student’s *t*-tests. Error bars indicate standard deviation. Wilcoxon’s tests were performed for (**D**–**G**,**I**).

The VAS values of pain in the buttocks and lower limbs were lower in group P only at the one-year time point (group R:P, n = 8:10; *p* = 0.033) (Figure 7E). All the other VAS values were similar between groups throughout the course, i.e., lower back pain (*p* = 0.41), pain in the buttocks and lower limbs (*p* = 0.28), and numbness in the buttocks and lower limbs (*p* = 0.84) at the baseline (group R:P, n = 7:11); lower back pain (*p* = 0.39) and numbness in the buttocks and lower limbs (*p* = 0.20) one year after surgery; lower back pain (*p* = 0.22), pain in the buttocks and lower limbs (*p* = 0.53), and numbness in the buttocks and lower limbs (*p* = 0.60) two years after surgery (group R:P, n = 7:8) (Figure 7A–D,F–I).

### 3.5. Assessments of Whether the Kissing Spine Contributes to the Radiographic and Clinical Outcomes

We hypothesized that the kissing spine may play an important role in the aforementioned radiographic and clinical outcomes by resecting the posterior elements. Therefore, we assessed whether the kissing spine contributes to the outcomes using a logistic regression analysis and a single regression analysis. Each parameter was discretely analyzed as the outcome in a logistic regression analysis or a single regression analysis, in which the kissing spine was set as the explanatory parameter.

The kissing spine was not a significant factor for any of the radiographic parameters, which were the 2-year KL score, D score, and DH, and the clinical parameters, which were the JOABPEQ score and pain VAS value. The *p*-values of each parameter were as follows: 2-year KL score, *p* = 0.48; 2-year D score, *p* = 0.48; 2-year DH, *p* = 0.77; 1-year lower back pain, *p* = 1.00; 1-year lumbar function, *p* = 0.68; 1-year walking ability, *p* = 0.53; 1-year social life function, *p* = 1.00; 1-year mental health, *p* = 0.76; 2-year lower back pain, *p* = 0.67; 2-year lumbar function, *p* = 1.00; 2-year walking ability, *p* = 0.70; 2-year social life function, *p* = 0.11; 2-year mental health, *p* = 0.70; 1-year lower back pain, *p* = 0.32; 1-year pain in the buttocks and lower limbs, *p* = 0.43; 1-year numbness in the buttocks and lower limbs, *p* = 0.62; 2-year lower back pain, *p* = 0.34; 2-year pain in the buttocks and lower limbs, *p* = 0.88; and 2-year numbness in the buttocks and lower limbs, *p* = 0.18.

## 4. Discussion

The present study suggested the potential role of the posterior elements in protecting the IVD from degeneration after lumbar spine laminoplasty. Similarly, the posterior elements were suggested to have roles in improving postoperative symptoms from multiple aspects one year after surgery and to improving walking ability until two years after surgery. The small numbers of cases for the analyses of postoperative symptoms limit the statistical power modestly; however, symptoms related to walking ability remained better in the group with preserved posterior elements throughout the course. The holistic results suggest meaningful roles of the posterior elements when preserved in lumbar spine laminoplasty.

To consider the mechanism of consequential disorders after the posterior elements were resected, we hypothesized that the kissing spine may be a critical factor for these poor outcomes. We hypothesized that the kissing spine, the impinging spinous processes, shared the load with the anterior column and facet joints and/or blocked the extension of the spine to limit the range of motion. If this hypothesis was correct, the cases with a kissing spine should have had a significant impact by resection of the posterior elements but not in the cases without a kissing spine. Unexpectedly, the kissing spine was not a significant factor for any of the outcomes in the group with resected posterior elements. We, therefore, considered that not the load sharing or extension block but the tension band function by the posterior elements acted as a physiological stabilizer of the surgical site, leading to better symptoms. Flexion–extension range of motion or incidence of instability were similar between groups. These results were probably caused by muscular defense because the serial radiographs were obtained by active motion by patients, and the results might have changed if stress tests were performed with the assistance of doctors similar to joint stress tests.

Surgical time was significantly longer in the group with preserved posterior elements, indicating a technical difficulty in preserving the construct in lumbar spine laminoplasty. However, the laboratory data relevant to the invasiveness of surgery did not differ between groups. These results can be interpreted as the level of elongation of surgical time by the construct preservation did not manifest any obvious detrimental effect.

Muscle-preserving interlaminar decompression (MILD) [10,11] may be a reasonable theory reported by Hatta et al. However, there is a concern about loosening and disrupting the integrity of the supra- and interspinous ligaments. If this procedure disrupts the ligament function, IVD degeneration can occur. The weak point of the study was that only the flexion–extension angle of the surgical site was evaluated concerning IVD degeneration [11]. A representative hallmark of IVD degeneration is the loss of DH due to cell loss and extracellular matrix degradation [12,13]. Therefore, they might have detected DH loss if it was measured. This study provides limited evidence due to the study design because it was a single-arm study. Contrarily, our study is double-arm and therefore clarifies the effect of posterior elements preservation.

The lumbar spinous process splitting technique contributed to mitigated paravertebral muscle degeneration [14,15], but the rationale is somewhat unclear because non-union of the spinous processes is highly prevalent (40–70%) [4,16], and the mechanism of why the multifidus muscle attaching to the floating bone retains its integrity is unknown. Notably, there is a contrary report on the protective effects of the spinous process splitting technique regarding functional outcomes, lower back pain, and leg pain [2]. Non-union of the spinous process was significantly associated with the ODI and levels of lower back pain and leg pain according to Kakiuchi and Fukushima [16]. Their results concur with ours, where the non-union of the spinous process and resection of the posterior elements were considered similar conditions regarding malfunction of the tension band structure, leading to compromised lumbar spine-related symptoms. Moreover, the spinous process splitting technique has downsides when the kissing spine and foraminal stenosis are involved [17].

Full-endoscopic spine surgeries (FESSs) are rapidly developing techniques due to their low invasiveness. However, FESS is a very different technique compared to open surgeries, which necessitates surgeons to train until it becomes practical. Endoscopes and additional devices are necessary, causing economic burden or limitations of the facilities available for this surgery. Considering the availability, laminoplasty with preserved posterior elements can be operated with a basic set of devices.

The significance of the present study is the clear-cut design, namely, the direct comparison of the groups with and without preservation of the posterior elements, and the suggestion of the effects of the preserved posterior elements on the IVD and postoperative symptoms. However, there are also limitations of the present study.

A limitation about the evaluation of active flexion–extension angles is that they were patient-generated, and muscular guarding could mask true instability. Passive stress radiographs should be included in future work. The major limitation of the present study is the limited number of cases. In particular, in the symptom-based disability and pain assessments, the number of cases was the least due to the inclusion criteria, one-level surgery, usage of the JOABPEQ, which is a quite new system, and sufficient follow-up term. This downside limits our study to being exploratory. Nonetheless, the results of the symptoms were in agreement with a previous study [16] with regards to the theory that impaired tension band structure of the posterior elements leads to compromised lumbar spine-related symptoms. This concordance supports our results as feasible despite the small number of cases. In addition, the minimal clinically important difference in the DH decrement, which is useful for sample-size calculation when designing studies, was still not established. We could have analyzed it if we had a large amount of JOABPEQ data, in which a 20% change in each score indicates statistical significance. Therefore, further studies with larger numbers of cases are important.

## 5. Conclusions

The preserved posterior elements were considered to have a protective role on the IVD in lumbar spine laminoplasty. In addition, they could possibly have positive effects on postoperative health status from multiple aspects, especially on walking ability, through stability at the surgical site by the tension band function and consequent IVD integrity. Our study provides a translational hypothesis to fill the gap of scientific knowledge in the course of “bench to bedside”.

## Figures and Tables

**Table 1:** Demographic background and surgical levels.

Variable	Group R (n = 38)	Group P (n = 47)	*p*-Value
Age (mean)	74.7	72.7	0.31
Sex: male (proportion)	25 (65.8%)	30 (63.8%)	1.00
Surgical level			
L1/2	1 (2.2%)	0 (0.0%)	0.06
L2/3	9 (20.0%)	2 (4.2%)	
L3/4	11 (24.4%)	12 (25.0%)	
L4/5	22 (48.9%)	33 (68.8%)	
L5/S	2 (4.4%)	1 (2.1%)	
Comorbidity			
Diabetes mellitus	12 (31.6%)	11 (23.4%)	0.47
Hypertension	18 (47.4%)	19 (40.4%)	0.66
Chronic kidney disease	3 (7.9%)	7 (14.9%)	0.50
Rheumatoid arthritis	1 (2.6%)	0 (0.0%)	0.45
Smoking history			
Smoker	24 (63.2%)	28 (59.6%)	0.82

## Data Availability

Dataset is available on request from the authors.

## Appendix A

The results of the Shapiro–Wilk test are stated in *p*-values as follows: age, R: 0.01, P: 0.79; hemorrhage, R and P: <0.0001; surgical time, R: 0.11, P: <0.0001; WBC, R: 0.34, P: 0.03; Hb, R: 0.15, P: 0.41; Alb, R: 0.03, P: 0.62; CRP, R: 0.0002, P: <0.0001; CPK, R: 0.04, P: 0.13; 2-year KL score increment, R and P: <0.0001; 2-year D score increment, R and P: <0.0001; 2-year DH decrement, R: <0.0001; P: 0.67; 2-year increase of FE angle, R: 0.28, P: 0.41; baseline lower back pain VAS, R: 0.24, P: 0.43; baseline lower limbs pain VAS, R: 0.80, P: 0.23; baseline lower limbs numbness VAS, R: 0.72, P: 0.09; 1-year lower back pain VAS, R: 0.07, P: 0.001; 1-year lower limbs pain VAS, R: 0.75, P: 0.001; 1-year lower limbs numbness VAS, R: 0.52, P: 0.0006; 2-year lower back pain VAS, R: 0.38, P: 0.005; 2-year lower limbs pain VAS, R: 0.66, P: 0.08; 2-year lower limbs numbness VAS, R: 0.35, P: 0.006.
